# Lifestyle Behaviors and Health-Related Quality of Life in Cancer Survivors: A Latent Class Analysis

**DOI:** 10.1177/10901981231203978

**Published:** 2023-10-13

**Authors:** Jenny L. Olson, David E. Conroy, Scherezade K. Mama, Kathryn H. Schmitz

**Affiliations:** 1 The Pennsylvania State University, College of Health and Human Development, University Park, PA, USA; 2 The Pennsylvania State University, College of Medicine, Hershey, PA, USA; 3 The University of Texas MD Anderson Cancer Center, Houston, TX, USA

**Keywords:** quality of life, multiple health behaviors, physical activity, healthy diet, sleep, smoking

## Abstract

Healthy lifestyle behaviors can improve health-related quality of life (HRQOL) in cancer survivors; but the combination of behaviors most important for HRQOL is not known. This study investigated the patterns of lifestyle behaviors among cancer survivors and differences in HRQOL between behavioral classes. Cancer survivors (*n* = 2,463) were invited to participate in a cross-sectional survey. Participants (*N* **=** 591) were predominately female (63%) and non-Hispanic White (90%). Survey items included self-reported physical activity, diet, smoking, sleep, HRQOL, and demographics. Behavioral classes were estimated by latent class analysis. Differences between classes were assessed by latent class regression. Compared with the “healthy lifestyles” class (higher probabilities of meeting aerobic/strength-based activity guidelines, high fruit/vegetable intake, and no sleep problems; 11% of sample), the “sleep and diet problems with inconsistent physical activity” class (higher probabilities of not meeting strength-based guidelines, low fruit/vegetable intake, some sleep problems; marginally higher probability of meeting aerobic guidelines; 41%) had poorer general and physical HRQOL. The “poor physical activity and diet” class (higher probabilities of not meeting aerobic/strength-based guidelines, low fruit/vegetable intake, and some sleep problems; 48%) had poorer general, physical, and mental HRQOL. Few participants exhibited healthy lifestyle patterns associated with HRQOL. The findings provide opportunities to develop differentiated multiple behavior-change interventions, targeted to two common patterns of behavior. A large subgroup of cancer survivors was susceptible to suboptimal physical activity and diet, warranting interventions exclusively targeting these behaviors. Another subgroup was susceptible to suboptimal physical activity, diet, and sleep, indicating interventions for this group should include strategies targeting these three behaviors.

## Introduction

Cancer-related death rates have declined steadily over the past three decades, with a large proportion of the 17 million cancer survivors in the United States expected to survive many years after diagnosis and treatment (American Cancer Society, 2022). The experience of cancer survivorship among these individuals is an important and enduring public health concern. Poor health-related quality of life (HRQOL) is common among cancer survivors (Weaver et al., 2012) and contributes substantially to the physical, psychosocial, and financial burden of cancer (Sitlinger & Zafar, 2018). Widespread adoption of healthy lifestyles could improve HRQOL among cancer survivors (Rock et al., 2020); however, participation in healthy lifestyle behaviors is low in this population (Tollosa et al., 2019).

It is recommended that cancer survivors undertake regular aerobic (i.e., ≥150 min/week of moderate-intensity, or 75 min of vigorous-intensity activity) and muscle-strengthening physical activity (i.e., ≥2 times/week), consume a diet high in fruit and vegetables (i.e., ≥5 cups/day) and low in fat (i.e., <30% kcal/day), avoid smoking, and get good sleep (Centers for Disease Control and Prevention, 2020; Clinton et al., 2020; Rock et al., 2020). These recommendations are consistent with the guidelines for Americans without cancer (Centers for Disease Control and Prevention, 2022; U.S. Department of Agriculture & U.S. Department of Health and Human Services, 2020; U.S. Department of Health and Human Services, 2018, 2023). Although cancer survivors are more likely to meet recommendations on smoking, physical activity, and body mass index (BMI) compared with those without a history of cancer, only 14% of cancer survivors meet the guidelines for aerobic and muscle strengthening physical activity, 15%–18% consume sufficient fruits and vegetables, and 29%–45% consume <30% of daily calories from fat, while 14% are current smokers (Arem et al., 2020; Blanchard et al., 2008; Coups & Ostroff, 2005). Poor sleep is common among cancer survivors, with frequent sleep disturbances experienced by more than half of long-term cancer survivors (Strollo et al., 2020). Behavioral interventions can be effective in improving engagement in healthy lifestyle behavior among cancer survivors (Amireault et al., 2018; Stacey et al., 2015). Most cancer survivors could benefit from efforts to promote one or more of these healthy lifestyle behaviors.

Meeting a greater number of recommendations for healthy lifestyle behaviors is associated with better HRQOL in cancer survivors (Blanchard et al., 2008). Therefore, it is important for cancer survivors to adopt multiple healthy lifestyle behaviors, rather than focusing on single health behaviors in isolation. However, adherence to multiple healthy lifestyle behaviors is low among cancer survivors (Tollosa et al., 2019), and individuals who undertake one unhealthy behavior are likely to adopt multiple unhealthy behaviors (Spring et al., 2015). For example, a study in central Pennsylvania revealed that 38.5% of rural cancer survivors were likely to be physically inactive and sedentary, have very low fruit and vegetable intake and excessive fat intake, and poor sleep (Olson et al., 2023). This highlights a need for effective strategies to support cancer survivors to adopt and maintain multiple healthy lifestyle behaviors for improved HRQOL.

Performing more health behaviors is associated with better HRQOL, but multiple behavior change can be burdensome for participants and becomes increasingly complex with additional behaviors (Prochaska et al., 2008; Spring et al., 2015). Furthermore, there is limited evidence for specific combinations of behaviors that are most important for HRQOL. Without this information, it is difficult to know which combinations of behaviors to prioritize for optimal improvement of HRQOL in cancer survivors. Identifying classes of behaviors to prioritize and target may lead to more efficient multiple health behavior-change interventions that minimize burden on cancer survivors (Spring et al., 2015).

Cancer survivors are a diverse population, including individuals of differing age, sex, race and ethnicity, socioeconomic status, employment status, and rurality (Miller et al., 2022; National Cancer Institute, 2022; Zahnd et al., 2018). Cancer survivors also have a broad range of health and cancer-related characteristics, such as differing cancer type, age at diagnosis, weight status, and co-morbidities. Lifestyle interventions are often targeted to sub-groups of cancer survivors with common socio-demographic or health-related characteristics, such as those with specific types of cancer, rural dwellers, specific racial or ethnic groups, or to those facing socio-economic disadvantage. Sub-groups of cancer survivors might also be distinguished by the patterns of lifestyle behaviors they exhibit, independent of socio-demographic or health-related characteristics. As distinct from variable-centered approaches, which focus on relationships between variables within an entire population, person-centered approaches focus on identifying sub-groups of individuals within a population (Howard & Hoffman, 2018). Approaches, such as latent class analysis (LCA), may be able to identify common patterns of lifestyle behaviors in cancer survivors. Knowledge of how lifestyle behaviors group into classes among cancer survivors can provide unique insight into the subgroups of this population most vulnerable to diminished HRQOL.

Common patterns of lifestyle behaviors among cancer survivors in the United States need to be investigated to identify the specific combination of lifestyle behaviors most important for HRQOL. Therefore, the aims of this study were to: (a) identify classes of lifestyle behaviors in cancer survivors; (b) examine the demographic, health, and cancer-related characteristics of sub-groups of cancer survivors most likely to exhibit each profile of lifestyle behaviors; and (c) investigate differences in HRQOL of cancer survivors between each behavioral class.

## Method

### Design

This study was a secondary analysis of data from the Cancer and Pennsylvania Behaviors and Lifestyle Epidemiology (CAPABLE) study (Bluethmann et al., 2020; Mama et al., 2020). CAPABLE was a cross-sectional survey of health behaviors and risk factors in cancer survivors living in Pennsylvania. The study was approved by the Pennsylvania State University Institutional Review Board (Study00005800). Data were collected between April and July 2017.

### Sample

Cancer survivors with a previous diagnosis of one of the five most common cancer types (i.e., breast, lung, colorectal, prostate, and gynecological; Siegel et al., 2022) were identified through the Pennsylvania Cancer Registry. Two thousand five hundred individuals from 28 counties in Central Pennsylvania serviced by the Penn State Cancer Institute were randomly selected and invited to participate (500 from each cancer type). Hispanic and Non-Hispanic Black individuals were oversampled by double, to ensure adequate representation of racial/ethnic minority groups. Equal numbers of male and female survivors of lung and colorectal cancers were sampled. Registrants with missing registry data, including age, race, and zip code, were excluded. Participants were eligible if they were: (a) diagnosed with one of the five cancer types; (b) documented in the Pennsylvania Cancer Registry between 2011 and 2015; (c) able to read, write, and speak English; and (d) at least 20 years old.

Potential participants were sent a recruitment letter describing the study. Those who did not formally opt out within 2 weeks were mailed consent and health information privacy forms and a paper-based questionnaire. If no response was received after an additional 4 weeks, a second copy of the letter and survey packet were mailed to participants. Consent was implied either by agreement to participate or return of the completed questionnaire.

### Measures

Survey items were based on items included in the Behavioral Risk Factor Surveillance System (BRFSS; Pierannunzi et al., 2013).

#### Lifestyle Behaviors

Physical activity was assessed with eight items from the 2015 edition of the BRFSS (Centers for Disease Control and Prevention, 2021). The items assess types, instances, and duration of physical activity performed over the past month. Responses were scored in accordance with the BRFSS physical activity scoring protocol (Centers for Disease Control and Prevention, 2008). Participant’s physical activity levels were classified in accordance with the 2008 Physical Activity Guidelines for Americans (i.e., “met” aerobic guidelines ≥150 min of moderate or vigorous equivalent aerobic activity/week; “did not meet” aerobic guidelines <150 min/week; “met” muscle strengthening guidelines ≥2 days/week of moderate/high intensity muscle strengthening activities involving all major muscle groups; “did not meet” muscle strengthening guidelines <2 days/week) (U.S. Department of Health and Human Services, 2002).

Dietary behavior was assessed with The Multifactor Screener: NHIS 2000 (National Cancer Institute, 2019). The 17 items capture the types and frequencies of foods consumed in the last month. Items were scored in accordance with Screener instructions. Predicted percentage energy intake from fat was classified in accordance with American Institute for Cancer Research (AICR) dietary guidelines (“acceptable” <30% kcal/day; “excessive” >30% kcal/day) (Clinton et al., 2020). It was not possible to categorize fruit and vegetable intake in accordance with the AICR dietary guidelines (i.e., 5 cups of fruits and vegetables daily; Clinton et al., 2020), as only 12 participants (2% of sample) met this guideline. Category selection was data-driven, to facilitate comparisons between participants (“relatively high” predicted daily servings of fruits and vegetables, excluding French fries ≥3 servings/day; “relatively low” <3 servings/day).

Smoking behavior was assessed with five items from the BRFSS questionnaires (Centers for Disease Control and Prevention, 2021). Smoking status was categorized as: “current smokers,” ‘former smokers,’ and those who had “never smoked.”

Sleep was assessed with a single item used periodically on BRFSS questionnaires between 1995 and 2010 (Centers for Disease Control and Prevention, 2017), which asks, “During the past 30 days, for about how many days have you felt you did not get enough rest or sleep?” Responses were classified into two categories (“some” sleep problems ≥1 day without sufficient rest or sleep; “none” = no sleep problems).

#### Demographic Characteristics

Participant age, race, ethnicity, and rurality were extracted from the Pennsylvania Cancer Registry. The questionnaire included items to assess sex, family income, and employment status.

#### Health and Cancer-Related Characteristics

Cancer type and age at diagnosis were identified through the Pennsylvania Cancer Registry. Questionnaire items assessed weight in pounds and height in meters or inches, which were used to calculate BMI = kg/m^2^. Items were also included in the questionnaire to assess history of chronic health conditions (e.g., heart, lung, or kidney disease; depression; arthritis; asthma). Responses were summed to calculate a composite score representing the number of comorbidities (score range 0–9).

#### Health-Related Quality of Life

HRQOL was assessed using the 12-item SF-12 (Ware et al., 1996). Summary scales representing physical health and mental health were calculated in accordance with the instrument’s scoring protocol (Ware et al., 1998). Possible scores on each subscale range from 0 to 100, with higher scores indicating better HRQOL. A single item on the scale was used to assess general health. This item is positively and significantly correlated with measures of overall HRQOL (Cunny & Perri, 1991). Responses were collected on a 5-point scale (1 = excellent to 5 = poor). The item was reverse coded, with higher scores represented better HRQOL.

### Analysis

Descriptive statistics were calculated in R (R Core Team, 2021) and RStudio Integrated Development for R (v1.4.1106). Means and standard deviations were calculated to describe continuous variables. Frequencies and percentages were used to describe categorical variables.

LCA was used to identify classes of lifestyle behaviors in cancer survivors. LCA facilitates the identification of latent (unobservable) sub-groups within a population based on a given set of predictor variables, as distinct from cluster analyses, which group cases with similar scores across predictor variables (Weller et al., 2020). Latent class regression was used to assess differences in characteristics of cancer survivors most likely to be allocated to each latent class. These analyses were conducted in SAS version 9.4 for Windows (SAS Institute Inc., Cary, NC, USA) using the PROC LCA procedure (Lanza et al., 2007). Aerobic and muscle-strengthening physical activity, fruit and vegetable intake, predicted percentage energy intake from fat, and sleep were coded as binary measures, and smoking status was coded into three categories, as described above. PROC LCA estimates parameters by maximum likelihood and treats missing values as missing at random. Missing items are replaced by a product of items observed for each participant (Lanza et al., 2007). Data from all lifestyle behaviors included missing values (i.e., aerobic activity = 76 [13%], muscle strengthening activity = 83 [14%], fruit and vegetable consumption = 26 [4%], predicted percentage energy from fat = 39 [7%], smoking status = 23 [4%], insufficient sleep or rest = 84 [14%]). LCA models with two, three, and four classes were compared to determine the optimal number of classes representing lifestyle behaviors among cancer survivors. Competing models were compared using Akaike’s Information Criteria and the Bayesian Information Criteria, with lower values signaling better model fit.

Each participant was assigned to the lifestyle behavior class for which they had the highest probability of membership. For regression models, continuous covariates were transformed to *z*-scores (*M* = 0, *SD* = 1) for interpretability, and categorical variables were coded as binary measures. Covariates representing demographic characteristics (i.e., sex, age, employment status, household income), health and cancer-related characteristics (i.e., cancer type, age at diagnosis, BMI, comorbidities), and HRQOL were individually added to the model to examine differences by lifestyle behavior class, reported as odds ratios and 95% confidence intervals (CIs). Covariates with missing values were omitted from the analysis (Lanza et al., 2007).

## Results

Descriptive statistics are presented in Table 1. Participants (*N* = 591; response rate = 24%) were aged between 25 and 95 years (*M* = 66.04 years; *SD* = 11.86). Most were female (63%), non-Hispanic White (90%), living in urban or metropolitan areas (90%), and retired (57%). Breast (25%) and gynecological (23%) cancers were the most common. On average, participants received a cancer diagnosis at age 64.54 years (*SD* = 11.75). Most were classified as overweight (30%) or obese (47%), and 72% reported at least one comorbidity. Scores on the HRQOL physical health subscale appeared low, relative to norms established in the general U.S. population (i.e., consistent with the 25th percentile of the general population) (Ware et al., 1998). Scores on the HRQOL mental health subscale appeared consistent with established norms in the general U.S. population (i.e., consistent with the 50th percentile of the general population). Due to the homogeneity in race, ethnicity, and rurality, these variables were excluded from further analyses.

**Table 1:** Participant Characteristics.

Demographic characteristics		*N*	%
Sex	Male	215	36.94
	Female	366	62.89
	Other	1	<1%
Race/Ethnicity	White non-Hispanic	533	90.19
	Minority	58	9.81
Family income	<$15,000/annum	71	12.2
	≥$15,000/annum	511	87.8
Rurality	Lives in a rural area	61	10.32
	Lives in a metropolitan area	530	89.68
Employment status	Not yet retired	147	43.11
	Retired	194	56.89
		*N*	*M (SD)*
Age	Years	577	66.04 (11.86)
Health and cancer-related characteristics		*N*	%
Cancer type	Breast	145	24.53
	Colorectal	108	18.27
	Gynecological	134	22.67
	Lung	81	13.71
	Prostate	123	20.81
		*N*	*M (SD)*
Age at diagnosis	Years	591	64.54 (11.75)
Body mass index	m/kg^2^	535	31.11 (8.29)
Comorbidities	Number of self-reported comorbidities	591	1.29 (1.07)
Health-related quality of life		*N*	*M (SD)*
Quality of life	General health (possible score range 0–5)	583	3.13 (0.91)
Quality of life	Physical health (possible score range 0–100)	541	43.75 (11.39)
Quality of life	Mental health (possible score range 0–100)	544	52.56 (8.05)

The number and proportion of participants classified in each category of lifestyle behavior are presented in Table 2. Half met the recommendations for aerobic physical activity (50%), while only a quarter met the recommendations for muscle-strengthening activity (25%). Similarly, around a quarter reported consuming three or more daily servings of fruit and vegetables (27%). Most participants reported one or more nights of insufficient sleep or rest over the past month (63%). An overwhelming majority reported predicted energy from fat greater than 30% (90%) and that they were not current smokers (92%). Due to the homogeneity in smoking and predicted fat intake, these variables were excluded from further analyses.

**Table 2.** Categories of Lifestyle Behaviors.

Lifestyle Behavior	Category	*N*	*%*
Guidelines for aerobic physical activity	Met	257	49.91
	Did not meet	258	50.10
	Missing	76	
Guidelines for muscle strengthening physical activity	Met	125	24.61
	Did not meet	383	75.39
	Missing	83	
Fruit and vegetable intake (daily)	Relatively high	151	26.73
	Relatively low	414	73.27
	Missing	26	
Predicted % energy from fat	Acceptable	56	10.14
	Excessive	496	89.86
	Missing	39	
Smoking status	Never	316	55.63
	Former	204	35.92
	Current	48	8.45
	Missing	23	
Days without enough sleep or rest in the past 30 days	None	188	37.08
	Some	319	62.92
	Missing	84	

Fit indices for the two, three, and four latent classes models are presented in Supplementary Table 1. The two-class model exhibited marginally better fit than the three-class model; however, the three-class model was the most interpretable and was chosen as the basis for subsequent analyses (Lanza et al., 2007). Latent class membership and lifestyle behavior response probabilities for the less interpretable two-class model are presented in Supplementary Table 2. In the two-class model, the first class (60% of participants) had greater probabilities of not meeting the guidelines for aerobic or strength-based physical activity. This class also had marginally higher probabilities of reporting some nights with insufficient sleep and relatively high fruit and vegetable intake. The second class (40% of participants) had higher probabilities of meeting guidelines for aerobic activity, relatively high fruit and vegetable intake, and reporting some nights with insufficient sleep. The second class also had a marginally higher probability of not meeting guidelines for strength-based physical activity.

**Figure 1.**
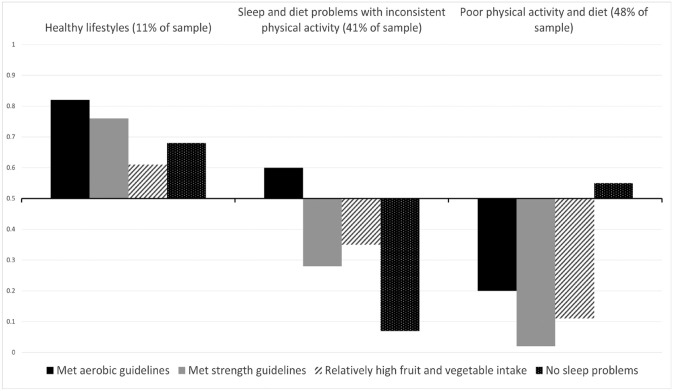
Response Probabilities for Healthy Behaviors for Each Behavioral Class.

Response probabilities for healthy lifestyle behaviors by behavioral class in the model selected for subsequent analysis are presented in Figure 1. Latent class membership and lifestyle behavior response probabilities for the model are presented in Supplementary Table 3. The first class (11% of participants) had greater probabilities of meeting the aerobic and strength guidelines for physical activity, relatively high fruit and vegetable intake, and of reporting no nights with insufficient sleep. This class was labeled “healthy lifestyles.” The second class (41% of participants) had high probabilities of reporting one or more nights of insufficient sleep, not meeting strength activity guidelines, and low fruit and vegetable intake. In contrast, this class also had somewhat higher probability of meeting the aerobic guidelines. The second class was labeled “sleep and diet problems with inconsistent physical activity.” The third class (48% of participants) had low probabilities of meeting guidelines for either aerobic or strength-based activity, and a high probability of low fruit and vegetable intake. This class was therefore labeled “poor physical activity and diet.”

Table 3 summarizes the odds of class membership for demographic, health, and cancer-related characteristics, and HRQOL. Relative to the healthy lifestyle class, those in the sleep and diet problems with inconsistent physical activity class had greater odds of higher BMI (OR = 3.14; 95% CI = [1.28, 7.72]) and comorbidities (OR = 2.21; 95% CI = [1.47, 3.33]). This class also had lower odds of being older (OR = 0.56; 95% CI = [0.35, 0.90]), being older at diagnosis (OR = 0.59; 95% CI = [0.36, 0.96]), and having better general health (OR = 0.24; 95% CI = [0.14, 0.42]) and physical health (OR = 0.42; 95% CI = [0.28, 0.64]).

**Table 3.** Odds of Class Membership for Demographic, Health, and Cancer-Related Characteristics, and for Health-Related Quality of Life.

Covariates	Odds ratio (95% CI)	
	Class 2 (*n* = 242; 41% of sample)	Class 3 (*n* = 284; 48% of sample)
	Sleep and diet problems with inconsistent physical activity	Poor physical activity and diet
Sex (male)^a^	2.21 (0.90–5.43)	1.13 (0.47–2.72)
Household income (≥$15,000/year)^b^	1.76 (0.35–8.86)	1.29 (0.55–3.02)
Employment status (retired)^c^	0.01 (0.00–2.89)	1.25 (0.24–6.50)
Age (years)	0.56 (0.35–0.90)^*^	1.74 (1.07–2.82)^*^
Cancer type (breast)^d^	1.33 (0.63–2.78)	2.15 (0.93–4.97)
Cancer type (colorectal)^d^	0.90 (0.19–4.37)	1.04 (0.44–2.44)
Cancer type (gynecological)^d^	0.96 (0.46–1.99)	0.11 (0.00–7.93)
Cancer type (lung)^d^	0.38 (0.06–2.28)	0.51 (0.20–1.29)
Cancer type (prostate)^* d^	0.63 (0.32–1.26)	2.06 (0.95–4.47)
BMI kg/m^2^	3.14 (1.28–7.72)^*^	0.58 (0.17–1.97)
Age at diagnosis (years)	0.59 (0.36–0.96)^*^	1.61 (1.05–2.45)^*^
Comorbidities (number)	2.21 (1.47–3.33)^*^	1.85 (1.29–2.66)^*^
Quality of life (general health)	0.24 (0.14–0.42)^*^	0.37 (0.23–0.59)^*^
Quality of life (physical health)	0.42 (0.28–0.64)^*^	0.47 (0.30–0.74)^*^
Quality of life (mental health)	2.56 (0.94–6.97)	0.18 (0.06–0.59)^*^

*Notes*: Reference group is Class 1, healthy lifestyles (*n* = 65; 11% of sample), ^*^p < .05. ^a^Compared with female and other; ^b^Compared with <$15,000/year; ^c^Compared with employed and unemployed/unable to work; ^d^Compared with all other cancer types.

When compared with the healthy lifestyles class, the poor physical activity and diet class had higher odds of being older (OR = 1.74; 95% CI = [1.07, 2.82]), being older at diagnosis (OR = 1.61; 95% CI = [1.05, 2.45]), and of having more comorbidities (OR = 1.85; 95% CI = [1.29, 2.66]). This class also had lower probabilities of reporting better general health (OR = 0.37, 95% CI = [0.23, 0.59]), physical health (OR = 0.47; 95% CI = [0.30, 0.74]), and mental health (OR = 0.18; 95% CI = [0.06, 0.59]).

## Discussion

Few cancer survivors in this study (11%) reported the pattern of healthy lifestyle behaviors (i.e., sufficient aerobic and muscle-strengthening physical activity, relatively high fruit and vegetable consumption, and no nights with insufficient sleep) associated with better HRQOL. Most (89%) exhibited one of two distinct patterns of less healthy behaviors. The low proportion of participants reporting a healthy pattern of lifestyle behaviors provides insight into why so many cancer survivors may be experiencing poor HRQOL and indicates a widespread need for healthy lifestyle behavior interventions among cancer survivors.

Healthy lifestyles incorporating a combination of aerobic and strength-based physical activity, higher daily fruit and vegetable intake, and an absence of sleep problems were associated with better physical, mental, and general HRQOL in cancer survivors. This finding is consistent with previous research. For instance, Blanchard et al. (2008) found that meeting individual recommendations for aerobic physical activity and fruit and vegetable consumption, along with avoiding smoking, was associated with better HRQOL among cancer survivors, and that meeting a greater number of behavioral recommendations overall was associated with better HRQOL. The non-significant difference in mental HRQOL between the healthy lifestyles and sleep and diet problems with inconsistent physical activity classes was counter to our hypotheses. The sleep and diet problems with inconsistent physical activity class had a marginally higher probability of meeting the guidelines for aerobic physical activity (.60). Physical activity has long been established as a correlate of better mental health (Taylor et al., 1985). Thus, it is possible that levels of aerobic activity in this class, although inconsistent, were sufficient to protect against diminished mental HRQOL, despite poor diet and insufficient sleep. The application of a person-centered methodological approach in the present study allows for the extension of previous findings by facilitating the identification of the specific patterns or combinations of lifestyle behaviors most important for HRQOL in cancer survivors. This combination of behaviors can be targeted in multiple behavior-change interventions aiming to improve HRQOL in cancer survivors.

The findings indicate a need to differentiate behavior-change strategies to suit the two less healthy profiles of lifestyle behaviors commonly exhibited by cancer survivors. The probability of not meeting strength guidelines and low fruit and vegetable intake was high in both less healthy classes, whereas there were divergent patterns of sleep and aerobic activity. Sleep provided a key differentiating factor between the groups, offering an opportunity for interventionists to quickly identify the most appropriate behavioral strategy to support individual cancer survivors. Multiple behavior-change interventions targeting improved sleep, diet, and physical activity are indicated for the large sub-group of cancer survivors with sleep and diet problems and inconsistent physical activity. Behavior-change interventions targeting physical activity and diet can support improved HRQOL in cancer survivors (Castro-Espin & Agudo, 2022; Mishra et al., 2014), while cognitive-behavioral therapy is recognized as an effective treatment for insomnia in cancer survivors. However, restricting the focus of interventions exclusively to physical activity and diet in the absence of sleep problems can ensure minimal intervention complexity and participant burden for almost half of cancer survivors, an important consideration for multiple behavior-change interventions (Prochaska et al., 2008; Spring et al., 2015).

Some of the characteristics of cancer survivors in each of the less healthy classes differed when compared with the healthy lifestyle class. Almost half of the cancer survivors in this study (48%) were classified in the poor physical activity and diet class. Members of this class were older, older when diagnosed with cancer, and were more likely to have comorbidities compared with the healthy lifestyle class. The population of cancer survivors in the United States is aging and the prevalence of comorbidities increases with age (Bluethmann et al., 2016). Comorbidities are associated with poorer HRQOL in cancer survivors (Fu et al., 2015; Vissers et al., 2013). Our findings suggest that older cancer survivors (i.e., those older than the sample mean age of 66 years) with comorbidities may also be at high risk of behavioral profiles incorporating unhealthy dietary and physical activity behaviors, but not necessarily poor sleep. This pattern of unhealthy energy balance behaviors in a vulnerable population of older adults with comorbidities likely increases the risks of other negative outcomes, such as cancer recurrence, premature mortality, and unhealthy weight gain (Rock et al., 2020). Thus, our research reinforces previous calls to prioritize the research agenda and intervention efforts to better support the growing population of older cancer survivors with comorbidities (Bluethmann et al., 2016). Such support may be provided in the form of multiple behavior-change interventions encouraging older cancer survivors with comorbidities to become more active and eat a healthier diet. Such interventions should include behavior-change techniques that are known to promote behavior change and maintenance in older adults, such as biofeedback, behavioral demonstration, practice/rehearsal, graded tasks, action planning, and prompts/cues for increasing physical activity (Howlett et al., 2018).

The other unhealthy class of behavior associated with poorer general health and physical health was the sleep and diet problems with inconsistent physical activity class (41% of participants). Members of this class were younger, had higher BMI, and more comorbidities than the healthy lifestyles class. Given positive energy balance (i.e., excess energy consumption over expenditure) contributes to weight gain, and high BMI is associated with sleep problems (Williams et al., 2015), it is unsurprising that members of this class were more likely to have higher BMI than the healthy lifestyle class. The probability of experiencing at least one night of insufficient sleep in this class was extremely high (i.e., 0.93). These findings build a further understanding of the known risks associated with poor sleep among cancer survivors (Gooneratne et al., 2007; Strollo et al., 2020), by showing that cancer survivors with comorbidities and unhealthy weight may be particularly vulnerable to experiencing insufficient sleep and relatedly, to diminished HRQOL. For this sub-group of cancer survivors, it appears critical to include strategies for improved sleep, such as the application of cognitive behavioral therapy, an established treatment for improving sleep in cancer survivors (Savard & Savard, 2017), within behavior-change interventions aiming to improve HRQOL.

Supporting cancer survivors to perform strength-based physical activity, in addition to aerobic activity, may also help to alleviate sleep problems in this population. A recent study by Dieli-Conwright et al. (2021) found that an intervention aiming to increase aerobic and strength-based physical activity in breast cancer survivors was associated with improved sleep quality. However, people with obesity face a range of physical, psychological, and external barriers to physical activity (McIntosh et al., 2016). Therefore, it is important that strategies designed to support the adoption and maintenance of aerobic and muscle-strengthening activities also include techniques to help cancer survivors with obesity to overcome these barriers.

The present study provides unique insight into patterns of health behaviors in cancer survivors, and some of the characteristics of cancer survivors likely to be included in each behavioral class. Aerobic and strength-based physical activity, fruit and vegetable intake, and sleep are associated with better HRQOL in cancer survivors and should be included in multiple behavior-change interventions to improve quality of life in this population. Public health resources should be prioritized for the development of sleep interventions for younger cancer survivors with unhealthy weight and comorbidities, and energy balance interventions for older cancer survivors with comorbidities. The sample was primarily White, non-Hispanic, and living in more urbanized areas. This precluded investigation of differences in these socio-demographic characteristics between behavioral classes. Similarly, homogeneity in smoking status and fat intake among participants precluded investigation of how these behaviors were grouped with other lifestyle behaviors. Future studies could extend the present findings by including cancer survivors from diverse racial and ethnic backgrounds, and those living in more diverse rural and metropolitan settings. The inclusion of additional lifestyle behaviors, such as alcohol intake and sedentary behavior could also extend the findings of the present work. The results of this study are based on data collected prior to the COVID-19 pandemic, which has affected the lifestyle behaviors of many (Knell et al., 2020; Zvolensky et al., 2020). The class structure and distribution of the health behaviors of cancer survivors may have shifted accordingly. The stage of cancer at diagnosis, the time elapsed since diagnosis, and the type of treatment received may also have influenced the lifestyle behaviors performed by cancer survivors; however, these constructs were not assessed, and findings should be interpreted accordingly.

The cut-points for the categorization of the two physical activity behaviors were informed by the 2008 Physical Activity Guidelines for Americans (U.S. Department of Health and Human Services, 2002), whereas selection of cut-points for daily fruit and vegetable intake were data-driven. Similarly, sleep was coded dichotomously, based on the presence (i.e., ≥ 1 days of insufficient sleep or rest over the past month) or absence of sleep problems. The adoption of alternative cut-points for the categorization of these behaviors could alter the results, and the findings should be interpreted accordingly. Furthermore, the limitations of self-reported measures of lifestyle behaviors are well known and the findings should be interpreted in the context of these limitations. Of the 2,463 cancer survivors invited to participate, 591 were included in the analyses. The 24% response rate is within the bounds of those observed for the BRFSS, depending on the state and survey year (19% and 62%) (U.S. Department of Health and Human Services, 2022); however, the moderate response rate should also be considered when interpreting the findings. Furthermore, no descriptive data were collected on non-responders, so we were unable to determine if there were differences between participants and those listed on the cancer registry who did not accept the invitation to take part. Finally, the cross-sectional design precludes causal inferences. For instance, it cannot be determined if health lifestyles cause improvements in HRQOL, or whether better HRQOL supports individuals to adopt healthy lifestyles. Intervention research could provide insight into causal relations between behavioral classes and HRQOL.

## Conclusion

Lifestyles incorporating higher levels of aerobic and strength-based physical activity, fruit and vegetable consumption, and no nights of insufficient sleep were associated with better physical, mental, and general HRQOL; however, this pattern of lifestyle behaviors was uncommon among the cancer survivors in this study. Most reported one of two patterns of less healthy lifestyle behaviors associated with poorer HRQOL. Almost half had very low probability of being physically active or having a healthy diet. Cancer survivors with this pattern of behavior were more likely to be older and to have comorbidities, compared with the healthy lifestyle class. Over 40% had poor diet and inconsistent physical activity, with a high probability of one or more nights of insufficient sleep. Members of this class were more likely to be younger and have a higher BMI and more comorbidities than the healthy lifestyle group. Distinct strategies targeting these specific combinations of behaviors, and the unique needs of the subgroups of cancer survivors likely to exhibit each profile of behaviors, are warranted.

## Supplemental Material

sj-docx-1-heb-10.1177_10901981231203978 – Supplemental material for Lifestyle Behaviors and Health-Related Quality of Life in Cancer Survivors: A Latent Class Analysis Supplemental material, sj-docx-1-heb-10.1177_10901981231203978 for Lifestyle Behaviors and Health-Related Quality of Life in Cancer Survivors: A Latent Class Analysis by Jenny L. Olson, David E. Conroy, Scherezade K. Mama and Kathryn H. Schmitz in Health Education & Behavior
