# Supplementary material for: Lifestyle Behaviors and Health-Related Quality of Life in Cancer Survivors: A Latent Class Analysis
Source: Health Educ Behav. 2023 Oct 13;51(3):341–51. doi: 10.1177/10901981231203978 (PMC11092290; doi:10.1177/10901981231203978)
Supplement: sj-docx-1-heb-10.1177_10901981231203978 – Supplemental material for Lifestyle Behaviors and Health-Related Quality of Life in Cancer Survivors: A Latent Class Analysis [file sj-docx-1-heb-10.1177_10901981231203978.docx]

**Lifestyle Behaviors and Health-Related Quality of Life in Cancer Survivors:**

**A Latent Class Analysis**

**Supplementary Table 1**

Latent class analyses fit statistics

| **Model** | **AIC** | **BIC** | **Adjusted BIC** |
| --- | --- | --- | --- |
| Two-class model | 31.52 | 70.96 | 42.39 |
| Three-class model | 29.12 | 90.47 | 46.02 |
| Four-class model | 38.08 | 121.33 | 61.01 |

**Supplementary Table 2**

Latent class membership and lifestyle behavior response probabilities of the less interpretable 2-class model.

| 2 Class model | | | Class 1 | Class 2 |
| --- | --- | --- | --- | --- |
|  |  |  |  |  |
| Latent Class Membership % | |  | 60.49% | 39.51% |
| Aerobic guidelines | Met | | .19 | .81 |
|  | Did not meet | | .81 | .19 |
| Strength guidelines | Met | | .09 | .44 |
|  | Did not meet | | .91 | .56 |
| Fruit & vegetable intake | Relatively high | | .55 | .84 |
|  | Relatively low | | .45 | .16 |
| Sleep problems | None | | .43 | .28 |
|  | Some | | .57 | .72 |

**Supplementary Table 3**

Latent class membership and lifestyle behavior response probabilities of the selected 3-class model

| 3 Class model |  | Class 1 | Class 2 | Class 3 |
| --- | --- | --- | --- | --- |
|  |  | Healthy lifestyles | Sleep and diet problems with inconsistent physical activity | Poor physical activity and diet |
| Latent Class Membership | | 11.35% | 40.61% | 48.04% |
| Aerobic guidelines | Met | .82 | .60 | .20 |
|  | Did not meet | .18 | .40 | .80 |
| Strength guidelines | Met | .76 | .28 | .02 |
|  | Did not meet | .24 | .72 | .98 |
| Fruit & vegetable intake | Relatively high | .61 | .35 | .11 |
|  | Relatively low | .39 | .65 | .89 |
| Sleep problems | None | .68 | .07 | .55 |
|  | Some | .32 | .93 | .45 |
